# Genomic insights into the clonal reproductive *Opuntia cochenillifera*: mitochondrial and chloroplast genomes of the cochineal cactus for enhanced understanding of structural dynamics and evolutionary implications

**DOI:** 10.3389/fpls.2024.1347945

**Published:** 2024-03-07

**Authors:** Jing Liu, Yuqing Feng, Cheng Chen, Jing Yan, Xinyu Bai, Huiru Li, Chen Lin, Yinan Xiang, Wen Tian, Zhechen Qi, Jing Yu, Xiaoling Yan

**Affiliations:** ^1^ Eastern China Conservation Centre for Wild Endangered Plant Resources, Shanghai Chenshan Botanical Garden, Shanghai, China; ^2^ Zhejiang Province Key Laboratory of Plant Secondary Metabolism and Regulation, College of Life Sciences and Medicine, Zhejiang Sci-Tech University, Hangzhou, China; ^3^ College of Life Science, Shanghai Normal University, Shanghai, China; ^4^ Animal Plant and Food Inspection Center of Nanjing Customs District, Nanjing, China

**Keywords:** Cactaceae, clonal reproduction, gene transfer, homologous recombination, mitochondrial genome, *Opuntia cochenillifera*, plant breeding, RNA editing

## Abstract

**Background:**

The cochineal cactus (*Opuntia cochenillifera*), notable for its substantial agricultural and industrial applications, predominantly undergoes clonal reproduction, which presents significant challenges in breeding and germplasm innovation. Recent developments in mitochondrial genome engineering offer promising avenues for introducing heritable mutations, potentially facilitating selective sexual reproduction through the creation of cytoplasmic male sterile genotypes. However, the lack of comprehensive mitochondrial genome information for *Opuntia* species hinders these efforts. Here, we intended to sequence and characterize its mitochondrial genome to maximize the potential of its genomes for evolutionary studies, molecular breeding, and molecular marker developments.

**Results:**

We sequenced the total DNA of the *O. cochenillifera* using DNBSEQ and Nanopore platforms. The mitochondrial genome was then assembled using a hybrid assembly strategy using Unicycler software. We found that the mitochondrial genome of *O. cochenillifera* has a length of 1,156,235 bp, a GC content of 43.06%, and contains 54 unique protein-coding genes and 346 simple repeats. Comparative genomic analysis revealed 48 homologous fragments shared between mitochondrial and chloroplast genomes, with a total length of 47,935 bp. Additionally, the comparison of mitochondrial genomes from four Cactaceae species highlighted their dynamic nature and frequent mitogenomic reorganizations.

**Conclusion:**

Our study provides a new perspective on the evolution of the organelle genome and its potential application in genetic breeding. These findings offer valuable insights into the mitochondrial genetics of Cactaceae, potentially facilitating future research and breeding programs aimed at enhancing the genetic diversity and adaptability of *O. cochenillifera* by leveraging its unique mitochondrial genome characteristics.

## 1 Introduction

The cochineal cactus (*Opuntia cochenillifera* (L.) Mill., Cactaceae), a succulent tree or shrub indigenous to Mexico, thrives primarily in desert or dry shrubland biomes. This species has gained global cultivation due to its extensive use in food, fodder, and medicinal applications for centuries (Russell and Felker, 1987; Anaya-Pérez, 2001; Lans, 2006; Barba et al., 2017; Kondo et al., 2023; Prisa, 2023). Notably, *O. cochenillifera* has been historically important as a host plant for the cochineal insect (*Dactylopius coccus*), a source of the red cochineal or carmine dye, extensively utilized as a natural colorant in food and cosmetics (Barba et al., 2017; Ramadan et al., 2021).

In *O. cochenillifera*, as in many *Opuntia* species, clonal reproduction is the predominant reproductive mode (Majure and Puente-Martinez, 2014). This strategy likely evolved in response to the arid conditions typical of its habitat, where sexual reproduction is energetically costly and often challenging (Mandujano et al., 2007; Wang et al., 2018). There are two forms exits: vegetative multiplication and apomixis. Apomixis is the production of seeds without previous fertilization (Asker and Jerling, 1992). In *Opuntia*, adventitious embryony is a common developmental pathway leading to apomixis (Majure and Puente-Martinez, 2014). Furthermore, the occurrence of apomixis is often associated with polyploidy, a condition that can indirectly establish an apomictic cytotype in new ecological niches by enhancing the plant’s adaptive potential (Hojsgaard and Hörandl, 2019). The most prevalent form of vegetative multiplication in *O. cochenillifera* is through cladode detachment (Majure and Puente-Martinez, 2014). This mode of reproduction offers significant advantages in population expansion, as the high frequency of multiplication in *Opuntia* can maintain specific genetic combinations, perpetuate hybrids, develop dense populations, and facilitate colonization of new localities (Majure and Puente-Martinez, 2014). It is noteworthy that both forms of asexual reproduction in *O. cochenillifera* enhance plant recruitment efficiency, exhibiting high success rates, particularly in vegetative multiplication (Majure and Puente-Martinez, 2014).

Clonal reproduction in *O. cochenillifera*, while advantageous for certain aspects of cultivation, may inadvertently impede selective breeding processes. This reproductive strategy results in progeny that are genetically identical to the maternal plant, thus limiting gene segregation and, consequently, the potential for genetic diversity (Almeida et al., 2022; Carra et al., 2023). As a species extensively cultivated for various applications, *O. cochenillifera*, along with other cacti, faces an urgent need for breeding advancements. These improvements are essential for developing high-yielding, quality varieties that can withstand the biotic and abiotic stresses prevalent in their production environments (Gentile and La Malfa, 2022; Carra et al., 2023). In the context of climate change, there is increasing interest in *Opuntia* for its potential strategic role in arid areas, leveraging its high water-use efficiency (Gentile and La Malfa, 2022; Jorge et al., 2023; Prisa, 2023). However, exploiting *Opuntia* germplasm for breeding is complex due to the high rate of apomixis, reducing the efficiency of generating novel variability via conventional breeding methods (Gentile and La Malfa, 2022).

Recent studies indicate that mitochondrial genome engineering could facilitate genetic breeding, especially in plants with high clonal reproduction. Advanced gene editing systems, such as mitoTALENs (Kazama et al., 2019; Arimura et al., 2020; Takatsuka et al., 2022), Golden Gate cloning system (Kang et al., 2021), and TALEN-GDM (Forner et al., 2022), offer potential for inducing stable, heritable mitochondrial mutations (Maliga, 2022). Given the high repair mechanisms and low mutation rate in plant mitochondria (Christensen, 2013; Kazama et al., 2019), these genetic variations can be effectively fixed and inherited. Moreover, mitochondrial genome information can serve as a uniparental marker, widely applied in species identification, phylogenetic reconstruction, and population genetic analysis (Sperisen et al., 2001; Galtier et al., 2009; Duminil and Besnard, 2021; Khachaturyan et al., 2023). However, knowledge about the mitochondrial genome within the Cactaceae, particularly *Opuntia*, remains limited, with no complete mitochondrial genome information reported to date.

Plant mitochondrial genomes (mtDNA) exhibit a suite of unique properties that distinguish them markedly from their mammalian counterparts. Notably, plant mtDNA is substantially larger, ranging from 10 to 600 times the size of mammalian mtDNA, yet it harbors only about 50% more genes (Kubo and Newton, 2008). This discrepancy is intriguing, considering that plant mtDNA retains the standard genetic code and exhibits a low divergence rate in terms of point mutations (Ghulam et al., 2015; Møller et al., 2021). However, it demonstrates high recombinational activity, a characteristic that contributes significantly to its complexity (Gualberto and Newton, 2017). While most reported plant mitochondrial genomes are circular, some mitochondrial genomes show the coexistence of linear, multi-branch and multi-ring structures (Kubo et al., 2000; Notsu et al., 2002; Handa, 2003; Ogihara et al., 2005). This diversity stems from the abundance of repeat sequences within the plant mitochondrial genomes. These repeats act as hotspots for both inter-molecular and intra-molecular recombination, leading to genome rearrangements and the formation of various isomeric forms (Cole et al., 2018). The frequency of recombination, mediated by these repeat sequences, is a key determinant of the predominant structural form of mitochondrial genomes and a major factor in the expansion of mitochondrial genomes in higher plants (Andre et al., 1992; Mower et al., 2012). Furthermore, recombination in plant mtDNA can create novel reading frames, leading to the production of cytoplasmic male sterility, a trait widely exploited in crop breeding (Gualberto and Newton, 2017; Tang et al., 2017; Kazama et al., 2019); Additionally, mitochondrial mRNA maturation in plants involves a uniquely complex set of activities, including processing, splicing, and editing at hundreds of sites (Small et al., 2020; Møller et al., 2021). The unique properties of plant mitochondria not only underscore their complexity but also highlight their flexibility and integral involvement in various critical processes within the plant cell, including photosynthesis, photorespiration, CAM and C4 metabolism, heat production, temperature regulation, stress resistance mechanisms, programmed cell death, and genomic evolution (Møller et al., 2021).

In this study, we aim to comprehensively analyze the mitochondrial genome of *O. cochenillifera* (cochineal cactus), focusing on its assembly, repetitive sequences, RNA editing events, chloroplast genome comparison, and phylogenetic relationships with related species. Our goal is to enhance understanding of its evolutionary dynamics, adaptability, and genetic diversity, providing valuable genomic insights for this clonally reproductive crop.

## 2 Materials and methods

### 2.1 *O. cochenillifera* DNA extraction and mitochondrial genome assembly

The *O. cochenillifera* plants were cultivated at Shanghai Chenshan Botanical Garden. High quality genomic DNA were isolated from stem epidermis using the modified CATB method (Arseneau et al., 2017). A sample of 100 mg from the *O. cochenillifera* epidermis was pulverized in liquid nitrogen, followed by the addition of 400 μL of buffer FP1 and 6 μL of RNase A; the mixture was vigorously shaken for 1 minute before being allowed to settle at room temperature for 10 minutes. Subsequently, 130 μL of buffer FP2 was incorporated, shaken for 1 minute, and then centrifuged at 12,000 rpm for 5 minutes to separate the supernatant. Isopropyl alcohol, amounting to 0.7 times the volume of the supernatant, was added, and after centrifugation at 12,000 rpm for 2 minutes, the supernatant was discarded, preserving the precipitate. The precipitate was then washed with 600 μL of 70% ethanol, shaken for 5 minutes, centrifuged at 12,000 rpm for 2 minutes, and the wash repeated once after discarding the supernatant to retain the precipitate. The lid was opened and inverted to allow the remaining ethanol to dry for 5 to 10 minutes. Finally, an appropriate volume of TE buffer was added, and the sample was heated in a 65°C water bath for 30 minutes, intermittently inverted to ensure dissolution, resulting in the DNA solution.

DNBSEQ and Nanopore platforms were used for sequencing. DNBSEQ sequencing and Oxford sequencing were performed by Wuhan Benagen Tech Solutions Company (http://en.benagen.com/). DNBSEQ sequencing data was sequenced using the DNBSEQ-T7,Guangdong, CHN, and Nanopore sequencing was performed by Oxford Nanopore GridION × 5 Oxford Nanopore Technologies, Oxford, UK. Flye software was used to perform *de novo* assembly of Oxford Nanopore long reads derived from *O. cochenillifera*. Results were visualized using Bandage software (Wick et al., 2015). The BLASTn program (Chen et al., 2015) was then utilized, with conserved mitochondrial genes from *Arabidopsis thaliana* chosen as query sequences, to identify contigs containing these conserved mitochondrial genes. The draft mitochondrial genome of *O. cochenillifera* was identified based on the assembled contigs. Subsequently, short and long reads were mapped onto these contigs using BWA (Burrows-Wheeler Aligner) software (Li, 2013) and SAMTools software (Li and Durbin, 2009), and all mapped reads were retained. Finally, a hybrid assembly was performed using Unicycler (Wick et al., 2017) using a combination of Illumina short reads and Nanopore long reads. GFA format files produced by Unicycler are visualized using Bandage software (Wick et al., 2015).

### 2.2 Annotation of the mitogenome of *O. cochenillifera*

As reference genomes for the protein-coding genes of the mitochondrial genome, we selected *Arabidopsis thaliana* (NC_037304) and *Liriodendron tulipifera* (NC_021152.1). The mitochondrial genome was annotated using the Geseq v2.03 (Tillich et al., 2017) and the tRNA and rRNA of the mitochondrial genome were annotated using the tRNAscan-SE v2.0.11 (Lowe and Eddy, 1997) and BLASTN v2.13.0 (Chen et al., 2015), respectively. Finally, we manually corrected annotation errors in each mitochondrial genome using the Apollo v1.11.8 (Lewis et al., 2002).

### 2.3 Relative synonymous codon usage

We utilized Phylosuite (Zhang et al., 2020) to extract the protein-coding genes (PCGs) from the genome. Subsequently, we employed MEGA v7.0.26 (Kumar et al., 2016) to conduct codon preference analysis on the protein-coding genes of the mitochondrial genome and calculate the Relative Synonymous Codon Usage (RSCU) values. An RSCU value>1 indicates that the codon was preferentially used by amino acids, whereas an RSCU value<1 indicates the opposite trend.

### 2.4 Analysis of repeat elements

We identified repeated sequence, including simple sequence repeats (SSRs), tandem repeat, and dispersed repeat, using the MISA v2.1 (Beier et al., 2017), TRF (Benson, 1999), and REPuter online servers (Kurtz et al., 2001), respectively. Subsequently, we visualized the results using Excel 2021 and the Circos v0.69-9 (Zhang et al., 2013). The comparative analysis of the SSRs composition and number of *O. cochenillifera* were conducted with other three related species available in Cactaceae, *Mammillaria huitzilopochtli* (OP081771), *Selenicereus monacanthus* (OQ835513), and *Pereskia aculeata* (ON496936.1). Origin software (Origin Lab Corp. v 8) was used to draw the chordal graph (May and Stevenson, 2009).

### 2.5 Identification of homologous sequences among organelle genomes

We assembled the chloroplast genome of *O. cochienllifora* using GetOrganelle and annotated the chloroplast genome using CPGAVAS2 (Shi et al., 2019). We corrected the annotation results of the chloroplast genome using CPGView (Liu et al., 2023). Finally, we analyzed homologous sequences using the BLASTN and visualized the results using Circos package.

### 2.6 Synteny and phylogenetic and analysis

Based on the BLAST program, we obtained BLASTN results for pairwise comparisons of each mitochondrial genome, retaining homologous sequences with lengths exceeding 500 bp as conservative collinear blocks for drawing the Multiple Synteny Plot. Utilizing sequence similarity, we employed MCscanX (Wang et al., 2012) to generate the Multiple Synteny Plot for *O. cochienllifora* in comparison with closely related species. According to the genetic relationship, we selected 31 related species and download their mitochondrial genomes (**Supplementary Table S1**), then used PhyloSuite (Zhang et al., 2020) to extract common genes, used MAFFT v7.505 (Katoh and Standley, 2013) to perform multiple sequence alignment analysis, and then phylogenetic analysis was performed using IQ-TREE v2 (Minh et al., 2020), and the results of phylogenetic analysis were visualized using iTOL v4 (Letunic and Bork, 2019).

### 2.7 RNA editing site prediction

We analyzed the sequences of all protein-coding genes (PCGs) encoded by the mitochondrial genome of *O. cochienllifora*. For the prediction of C-to-U RNA editing sites within these mitochondrial PCGs, we employed Deepred-mt t (Edera et al., 2021), a tool based on a Convolutional Neural Network (CNN) model. This approach provided enhanced accuracy over previous prediction methodologies. We considered predictions with probability values exceeding 0.9 to ensure high confidence in our results.

## 3 Results

### 3.1 Characteristics of the *O. cochienllifora* mitogenome

**Figure 1:**
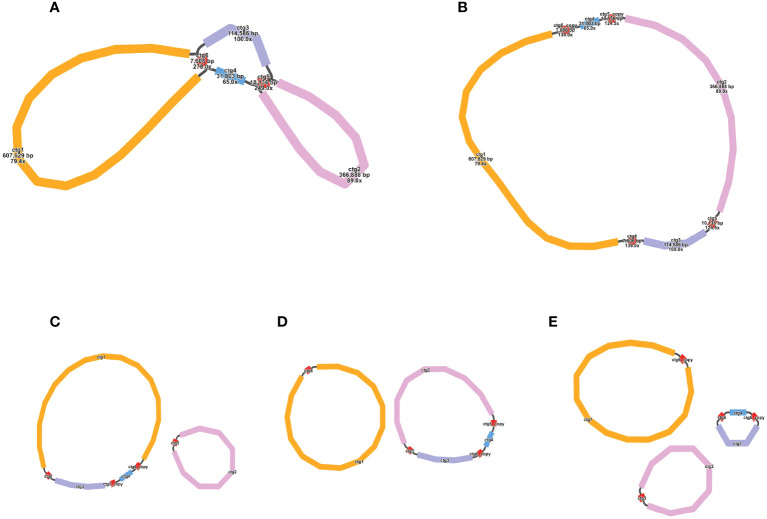
Potential isomers of *O. cochienllifora* mitogenome inferred from shorts reads and long reads. The initial assembly is shown in the Panel **1A**, with **(B–E)** representing the four possible isomers formed after solving the paths of the two pairs of repeating regions (ctg 5 and ctg 6). These isomers contain one “master ring” structure **(B)** that differ in sequence order, as well as one structure of two independent small rings **(C, D)** and one structure of three independent small rings **(E)**. The structure shown in panel **(B)** for the downstream analysis, which is supported by most long reads.

We used the Bandage to visualize the sketch of the mitochondrial genome assembled based on long-reads. The final result was depicted in **Figure 1A**, which comprises six nodes, each labeled with a specific name (refer to the graph1.gfa file for details). Detailed information about nodes was shown in **Table 1**. Each node represents a contig obtained through assembly. If two nodes are mutually connected by a black line, it signifies an overlap between the two sequences. All of these sequences collectively form a complex multi-branched closed genome structure, representing the complete mitochondrial genome sequences of *O. cochienllifora*. For critical nodes with branching, we resolved them using long-reads. We exported the relevant sequences at the branching nodes and mapped them to the long-reads. When two sequences connected by a black line appeared consecutively on the same long-read, it indicated that the long-read supported the connection between these two sequences. In cases where there were multiple potential connections at branching nodes, we prioritized connections that received greater support from long-reads. Red nodes represent potential repetitive sequences that occur multiple times in the genome. The sequence of a circular ‘master circle’ obtained after solving the branch nodes caused by repeated sequences (red nodes) based on long-reads data is shown in **Figure 1B**. The specific resolution path representing its master circle structure can be found in **Table 2**. Additionally, beneath the connections of two pairs of repetitive sequences, potential rearrangement configurations may exist, resulting in the genome splitting into multiple smaller circles (**Figures 1C, D**).

**Table 1:** Length and sequencing depth of each node.

Contig/Node	Length(bp)	Depth (×)
1	607,629	79.4
2	366,888	89.0
3	114,586	100.0
4	31,803	65.0
5	10,136	249.0
6	7,606	278.0

**Table 2:** Path selection for each node (repeating area) based on Nanopore data.

Contig	Type	Path
1	circular	ctg1-ctg6-ctg3-ctg5-ctg2-ctg5_copy-ctg4-ctg6_copy

### 3.2 Assembly and annotation of the mitochondrial genome of *O. cochienllifora*

**Figure 2:**
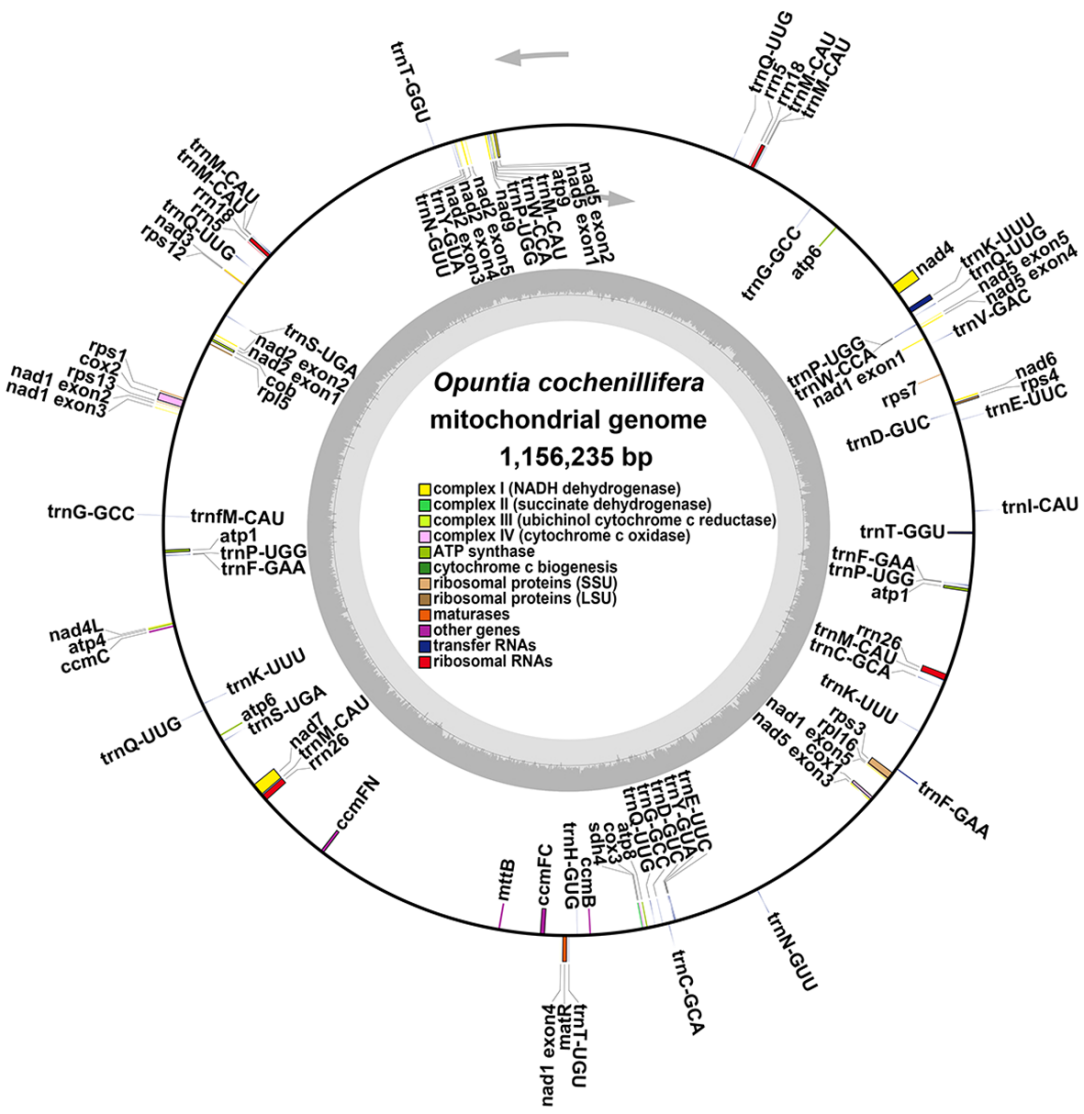
The putative circular mitogenome maps of *O. cochienllifora*. The genomic features inside and outside the circle represent the clockwise and counterclockwise chains on the transcription, respectively. Different color blocks represent different functional gene groups.

The main structure of the mitochondrial genome of *O. cochienllifora* was a single circular molecule. After excluding repetitive regions through ONT data, we obtained a mainly circular contig with a total length of 1,156,235 bp and a GC content of 43.06% (**Figure 2**, **Table 3**). The mitochondrial genome of *O. cochienllifora* was annotated, and a total of 33 unique mitochondrial protein-coding genes were annotated, including 24 core genes and nine non-core genes, 19 tRNA genes (of which 14 tRNAs were multi-copy), three rRNA genes (three of which had multiple copies of rRNA) (**Table 4**). The core genes included five ATP synthase genes (*atp1, atp4, atp6, atp8* and *atp9)*; nine *NADH* dehydrogenase genes (*nad1, nad2, nad3, nad4, nad4L, nad5, nad6, nad7* and *nad9*); four cytochrome C biogenesis genes (*ccmB, ccmC, ccmFC* and *ccmFN*); three cytochrome C oxidase genes (*cox1, cox2* and *cox3*); one membrane transport protein gene (*mttB*); one mature enzyme gene (*matR)* and one ubiquinol-cytochrome C reductase gene (*cob*). Non-core genes included two ribosomal large subunit genes (*rpl5, rpl16*); six ribosomal small subunit genes (*rps1, rps3, rps4, rps7, rps12, rps13*); one succinate dehydrogenase gene (*sdh4*). Analysis of repeat elements.

**Table 3:** Basic information of the mitochondrial genome of *O. cochienllifora*.

Type	Mitochondrial genome
Structure	Circular
Circular molecular	1
Total length	1,156,235bp
GC content	43.06%

**Table 4:** Information on the mitochondrial genome of *O. cochienllifora*.

Group of genes	Name of genes
ATP synthase	*atp1* (×2), *atp4*, *atp6* (×2), *atp8*, *atp9*
NADH dehydrogenase	*nad1*, *nad2*, *nad3*, *nad4*, *nad4L*, *nad5*, *nad6*, *nad7*, *nad9*
Cytochrome *b*	*cob*
Cytochrome *c* biogenesis	*ccmB*, *ccmC*, *ccmFC*, *ccmFN*
Cytochrome *c* oxidase	*cox1*, *cox2*, *cox3*
Maturases	*matR*
Protein transport subunit	*mttB*
Ribosomal protein large subunit	*rpl5*, *rpl16*
Ribosomal protein small subunit	*rps1*, *rps3*, *rps4*, *rps7*, *rps12*, *rps13*
Succinate dehydrogenase	*sdh4*
Ribosome RNA	*rrn5* (×2), *rrn18* (×2), *rrn26* (×2)
Transfer RNA	*trnC-GCA* (×2), *trnD-GUC* (×2), *trnE-UUC* (×2), *trnF-GAA* (×3), *trnfM-CAU*, *trnG-GCC* (×3), *trnH-GUG*,*trnI-CAU*, *trnK-UUU* (×3), *trnM-CAU* (×7), *trnN-GUU* (×2), *trnP-UGG* (×4), *trnQ-UUG* (×5), *trnS-UGA* (×2), *trnT-GGU* (×2), *trnT-UGU*, *trnV-GAC*, *trnW-CCA* (×2), *trnY-GUA* (×2)

### 3.3 Analysis of repeat elements

**Figure 3:**
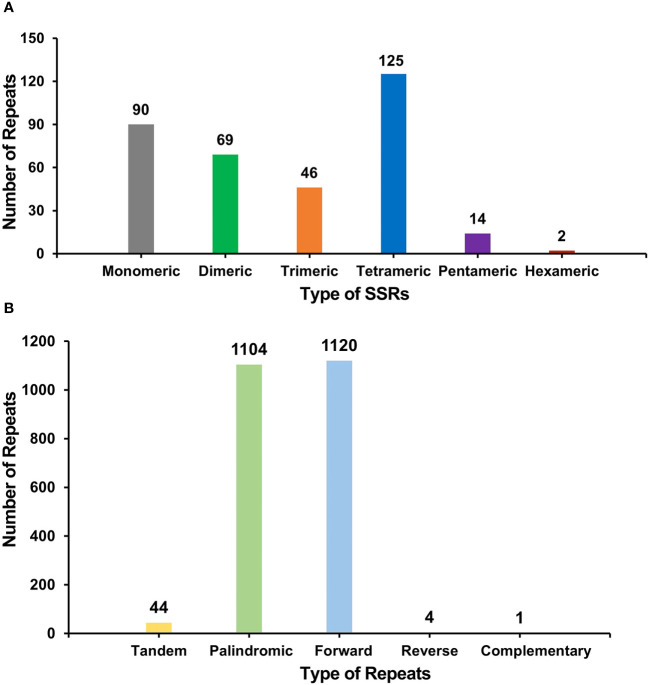
The SSRs and dispersed repeats identified in the mitogenomes of *O. cochienllifora*. **(A)** The SSRs identified in the *O. cochienllifora* mitogenomes. Each column represents different nucleotide repeated units displayed in different colors. **(B)** Dispersed repeats (≥30 bp) identified in the *O. cochienllifora* mitogenomes.

In the *O. cochienllifora* mitogenome, several repetitive sequences were observed (**Figure 3A**). A total of 346 SSRs were identified (**Figure 3A**, **Supplementary Table S2**). Monomeric and dimeric forms of SSRs accounted for 45.95% of the total SSRs. Adenine (A) monomer repeat accounted for 50.00% (45) of 90 monomer SSRs. We identified 44 tandem repeat sequences with a similarity greater than 69% and lengths ranging from 10 to 57 bp (**Supplementary Table S3**). The detection of dispersed repeat revealed a total of 2,229 pairs of repeat sequences with a length greater than or equal to 30 bp (**Supplementary Table S4**). Among these, there were 1,104 pairs of palindromic repeats, 1,120 pairs of forward repeats, 4 pairs of reverse repeats, and 1 pair of complementary repeats (**Figure 3B**). The longest palindromic repeat observed was 349 bp, while the longest forward repeat was 13,272 bp. The comparative analysis of the SSRs revealed that *O. cochienllifora* exhibited the highest number of unique SSRs (85), while *M. huitzilopochtli* only had 37 (**Supplementary Figure S2**, **Supplementary Table S5**). Among the species, *S. monacanthus*, *O. cochenillifera* and *P. aculeata* showed a high number of Tetra repeats (**Supplementary Figure S3**, **Supplementary Table S6**). Dispersed repeats were found to be prevalent in all four species (**Supplementary Figure S4**, **Supplementary Table S7**).

### 3.4 Codon usage analysis of PCGs

**Figure 4:**
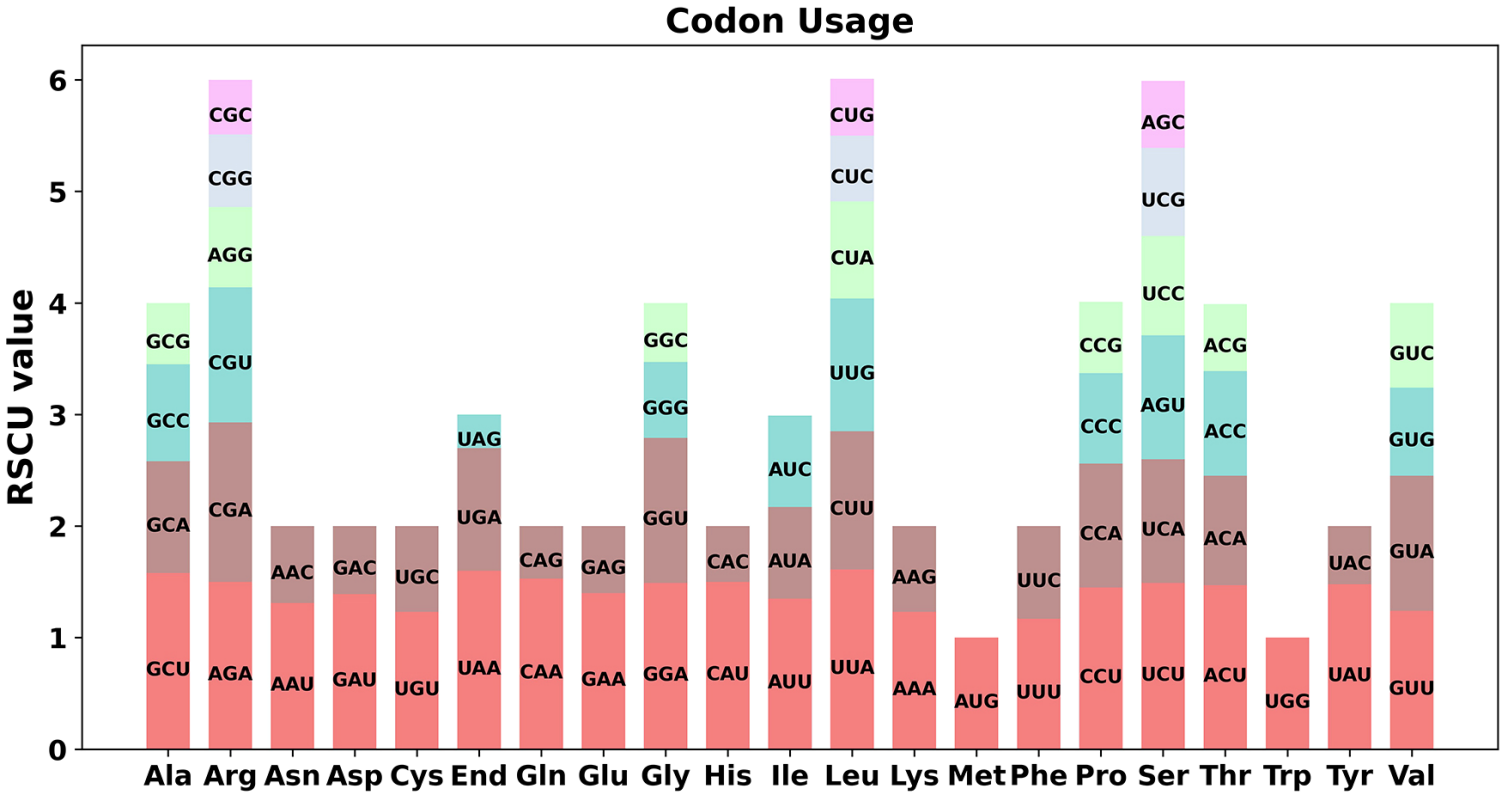
Codon usage bias of mitochondrial PCGs of *O. cochienllifora*. The RSCU refers to relative synonymous codon usage.

Codon preference analysis was performed on 33 unique protein-coding genes (PCGs) in the mitochondrial genome of the *O. cochienllifora.* The usage of each codon for amino acids was shown in **Supplementary Table S8**. Codons with a relative synonymous codon usage (RSCU) value greater than 1 were considered to be preferentially utilized for corresponding amino acids. As shown in **Figure 4**, aside from the start codon AUG and the Tryptophan codon (UGG), both with an RSCU value of 1. There was a general codon usage preference in mitochondrial PCGs. For example, Leucine had a high preference for UUA, with the highest RSCU value among mitochondrial PCGs at 1.61. Additionally, the stop codon UAA also showed a preference, with an RSCU value of 1.6.

### 3.5 Identification of homologous sequences among organelle genomes

**Figure 5:**
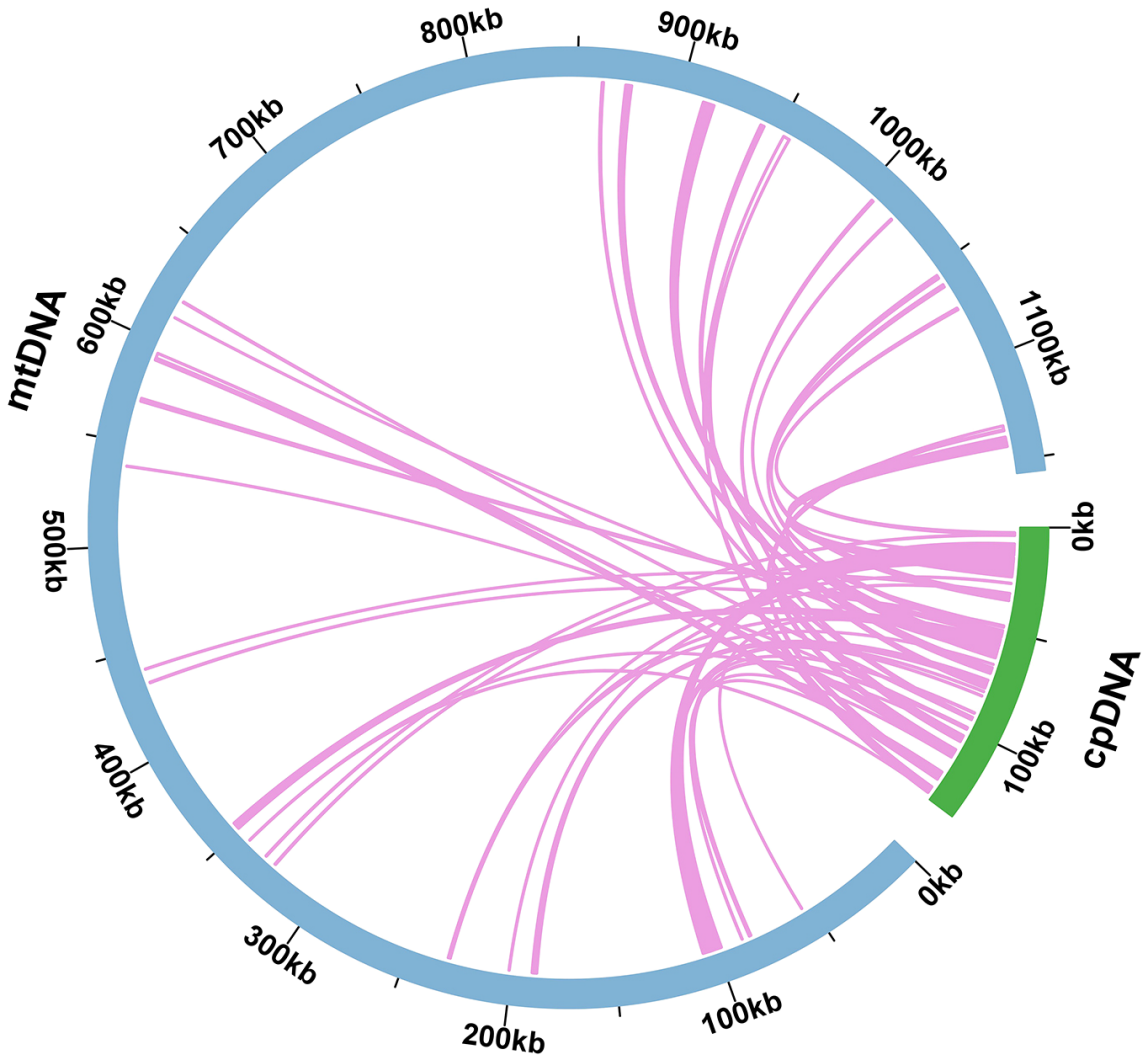
Schematic representation of homologous sequences between chloroplast genome and mitogenomes in *O. cochienllifora*. The blue arcs represent mitogenomes, the green arcs represent chloroplast genomes, and the lines between arcs correspond to homologous genome segments.

Mitochondrial plastid DNAs (MTPTs) were plastid-derived DNA fragments found in the mitochondrial genome. In the present study, sequencing data were used to assemble the *O. cochienllifora* plastome, which was 138,084 bp in size (**Supplementary Figure S1**). Based on the analysis of sequence similarity, a total of 48 MTPTs were identified in the *O. cochienllifora* plastome (**Figure 5** and **Supplementary Table S9**), with a total length of 47,935 bp, accounting for 4.15% of the mitogenome length and 34.71% of the total plastome. There were 19 fragments with lengths greater than 1,000 bp (**Supplementary Table S9**), of which MTPT5 was the longest at 5,450 bp. Through annotation of these homologous sequences, 35 complete genes were identified on the 48 homologous segments, including 21 PCGs (*atpH, matK, ndhF, ndhH, petA, petG, petL, petN, psaJ, psbD, psbE, psbF, psbJ, psbL, psbM, psbZ, rpl20, rpl33, rpoC2, rps15, rps16*) and 14 tRNA genes (*trnC-GCA, trnD-GUC, trnE-UUC, trnfM-CAU, trnG-GCC, trnI-CAU, trnK-UUU, trnM-CAU, trnN-GUU, trnP-UGG, trnQ-UUG, trnT-GGU, trnW-CCA, trnY-GUA*).

### 3.6 Phylogenetic analysis and synteny analysis based on mitochondrial genomes of higher plants

**Figure 6:**
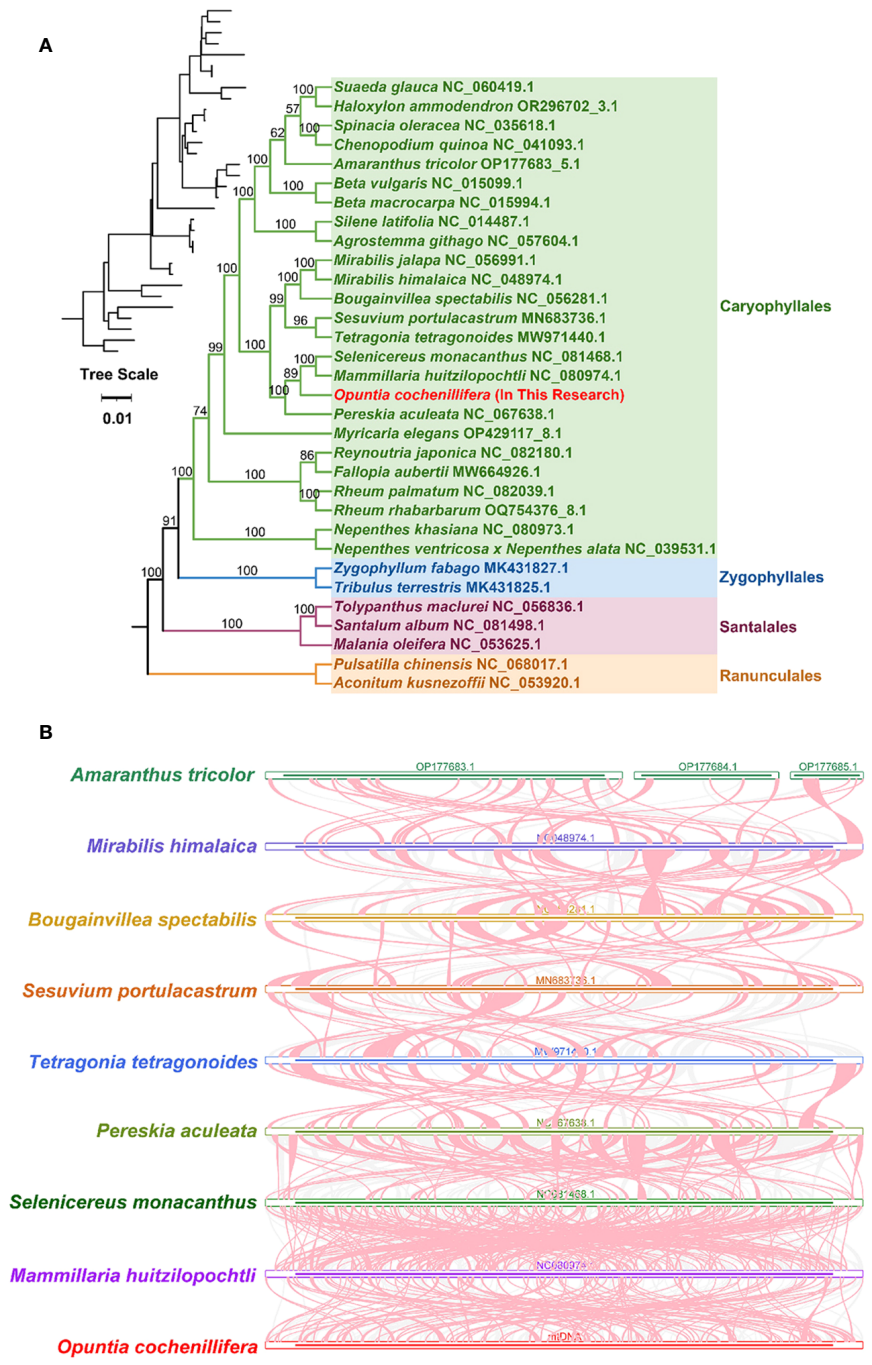
Phylogenetic and synteny analyses of *O. cochienllifora*. **(A)** The plants in the diagram belong to of Caryophyllales. Different families are represented by different colors, with *O. cochienllifora* represented in red. **(B)** Synteny analysis of nine mitogenomes. Only collinear blocks over 0.1 kb in length are retained. Red-curved regions indicate where inversions occur, gray regions indicate regions of good homology, and white regions indicate species-unique sequences.

A phylogenetic analysis was performed with 32 species based on the DNA sequence of 24 conserved mitochondrial PCGs (*atp*1, *atp*4, *atp*6, *atp*8, *atp*9, *ccmB*, *ccmC*, *ccmFC*, *ccmFN*, *cob*, *cox*2, *cox*3, *matR*, *nad*1, *nad*2, *nad*3, *nad*4*L*, *nad*5, *nad*6, *nad*7, *nad*9, *rpl*5, *rps*3, and *rps*12). Two mitochondrial genomes *Pulsatilla chinensis* (NC068017.1) and *Aconitum kusnezoffii* (NC053920.1) from the Ranunculales order were set as outgroups. The results showed that *O. cochienllifora* belonged to the Cactaceae family and was closely related to *S. monacanthus*, *M. huitzilopochtli*, and *P. aculeata* (**Figure 6A**). The topology of this mitochondrial DNA-based phylogeny was consistent with the latest classification of APG (Angiosperm Phylogeny Group).

Collinearity blocks with a length of less than 0.5 kb were excluded from the results. Extensive homologous collinearity blocks were identified between *O. cochienllifora* and closely related species in the Caryophyllales (**Figure 6B**, **Supplementary Table S10**). Additionally, some regions were found to be unique to *O. cochienllifora*, lacking homology with the rest of the species. The results indicated that the arrangement of collinearity blocks among the mitochondrial genomes of these nine species was inconsistent. The mitochondrial genome of *O. cochenillifera* exhibited a notable degree of genome rearrangements when compared with its closely related species within the Caryophyllales order. This was particularly evident in the mitochondrial genome sequences of the four cacti species, demonstrating extremely non-conservative arrangements and frequent genome recombination (**Figure 6B**).

### 3.7 The prediction of RNA editing events

**Figure 7:**
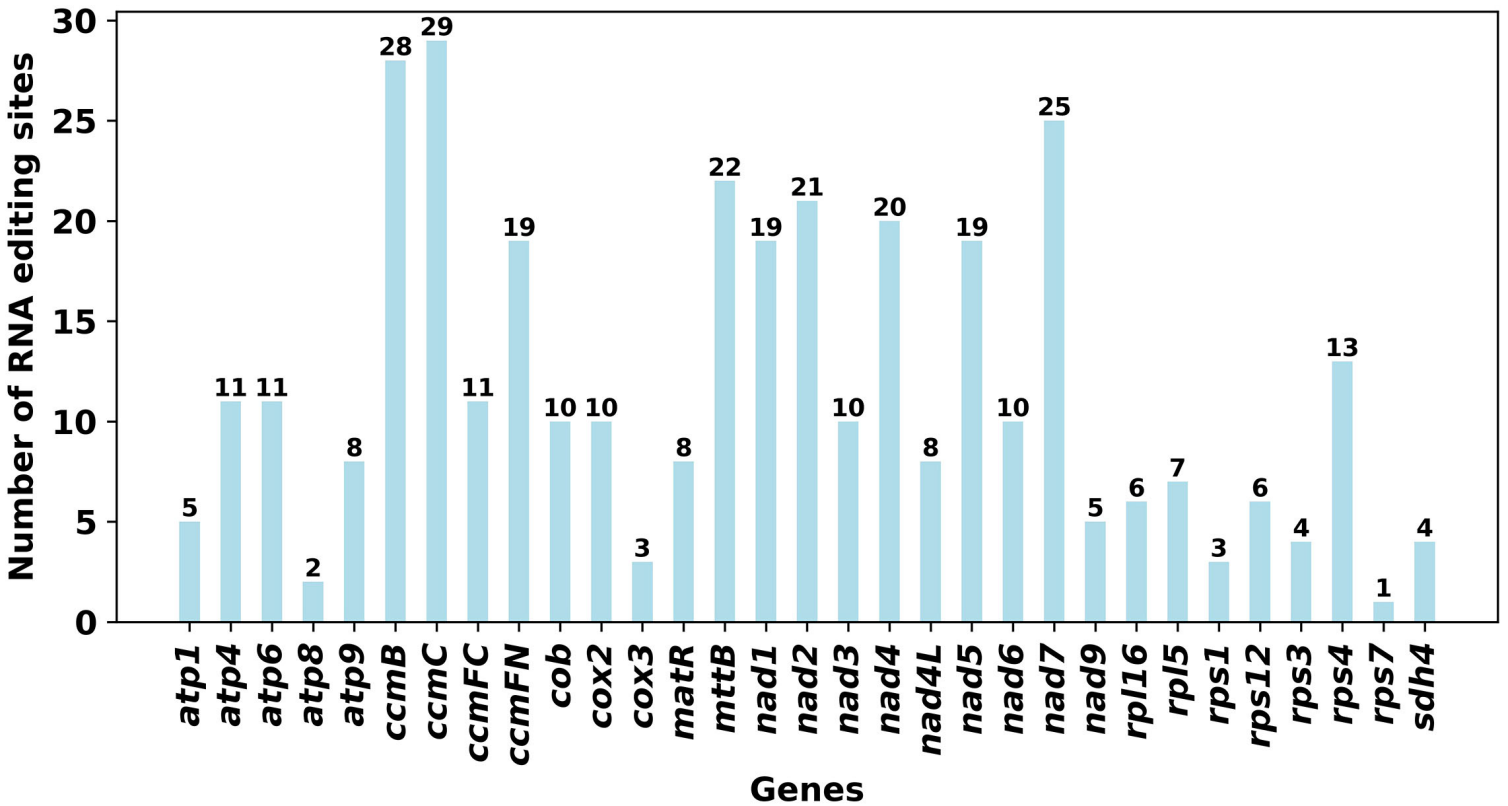
Characteristics of the RNA editing sites identified in PCGs of *O. cochienllifora* mitogenome. Number of RNA editing sites predicted by individual PCGs using Deepred-mt. The abscissa shows the name of the gene, and the ordinate shows the number of edited sites.

RNA editing events of 33 unique PCGs from *O. cochienllifora* mitochondrial genome were characterized. The cutoff value for identification was set at 0.9. Under this criterion, a total of 358 potential RNA editing sites were identified across the 33 mitochondrial PCGs, all of which were base C to U editing (**Figure 7**, **Supplementary Table S11**). Among the mitochondrial genes, 29 RNA editing sites were identified in the *ccmC* gene, which had the highest number of edits among all mitochondrial genes. Following closely was the *ccmB* gene, with 28 RNA editing events. We identified that the initiation codons of three genes (*cox2, nad4L*, and *nad7*) and termination codons of three genes (*atp6, atp9*, and *ccmFC*) were products of RNA editing events, and these were confidently verified by Deepred-mt.

## 4 Discussion

### 4.1 Size and genetic composition properties of the *O. cochienllifora* mitogenome

This study utilized a hybrid assembly strategy, combining short and long reads, to assemble the high-quality, full-length (1,156,235 bp) ring-like mitochondrial genome of *O. cochienllifora*. Compared to other species in the Cactaceae family, it was significantly larger than the mitochondrial genome of *P. aculeata* (515.2 kb) (Zhang et al., 2023), yet approximately half the size of *M. huitzilopochtli* (2.052 Mb) and *S. monacanthus* (2.290 Mb) (Lu et al., 2023; Plancarte and Solórzano, 2023). Previous research indicated that total genome size did not correlate with structural complexity (such as chromosome arrangement), gene count, gene identity, or GC content in plant mitochondrial genomes (Plancarte and Solórzano, 2023). The GC content of the *O. cochienllifora* mitochondrial genome was 43.06%. Although mitochondrial genome sizes vary greatly within the family, the GC content was remarkably consistent (43%-44.05%). The consistency in GC content across Cactaceae mitochondrial genomes might suggest a parallel evolutionary history among these species (Landrum, 2002; Copetti et al., 2017), as GC content diversity typically reflects adaptive consequences (Lassalle et al., 2015; Trávníček et al., 2019). Beyond its primary configuration, the *O. cochienllifora* mitochondrial genome exhibited alternative chromosomal structures (**Figure 1**), a characteristic also observed in various terrestrial plants (Whelan and Murcha, 2015; Gualberto and Newton, 2017; Møller et al., 2021).

### 4.2 Repeated sequences and extensive homologous recombination in the *O. cochienllifora* mitogenome

Repetitive sequences, which were found abundantly in mitochondrial genomes, played a crucial role in shaping the evolutionary landscape of plant adaptation, regulating gene expression, and influencing the variability of epistatic traits (Mehrotra and Goyal, 2014; Wynn and Christensen, 2019; Xiong et al., 2022). Within the mitochondrial genome of *O. cochienllifora*, our analysis identified a total of 346 simple sequence repeats (SSRs), forming a substantial collection of reference loci. These SSRs not only held potential for species identification but also served as valuable genetic markers in the exploration of *Opuntia* germplasm. This discovery implied that dispersed repeats may play a pivotal role in genome expansion and gene regulation (**Supplementary Figure S4**) (Gualberto and Newton, 2017). Furthermore, the presence of repetitive sequences in plant mitochondrial genomes has been associated with homologous repair mechanisms, which were integral to genome evolution and variation (Knoop, 2012; Christensen, 2013). Synteny analysis conducted in this study revealed significant recombination events within the mitochondrial genome, as evidenced by the remarkable shuffling of homologous regions among the four Cactaceae genera (**Figure 6B**). This observed phenomenon suggested a widespread evolutionary mechanism contributing to plant adaptation under stressful environmental conditions within the family (Hernández-Hernández et al., 2014; Copetti et al., 2017). The dynamic nature of the mitochondrial genome, shaped by repetitive elements and recombination, highlighted its pivotal role in the adaptation and evolution of plant species, particularly within the Cactaceae family.

### 4.3 Integration and potential functional implications of chloroplast-derived DNA in the mitochondrial genome of *O. cochienllifora*

Plant mitochondrial genomes, due to their unique structural and evolutionary characteristics, were more receptive to foreign DNA integration (Wynn and Christensen, 2019). It had been frequently observed that plant mitochondrial genomes incorporate DNA sequences of plastid origin (Wang et al., 2007; Alverson et al., 2011; Gao et al., 2020). In the mitochondrial genome of *O. cochienllifora*, homologous segments with the chloroplast genome spanned 47,935 bp, constituting 35% of its total chloroplast genome length. This significant proportion of chloroplast-derived segments, also noted in the *S. monacanthus* mitogenome (Lu et al., 2023), was a rare occurrence in both angiosperms and gymnosperms. Typically, these homologous fragments transferred several photosynthesis-related protein-coding genes (PCGs) to the mitochondrial genome (Alverson et al., 2011). Our data revealed that at least 21 intact PCGs, one of the highest numbers recorded, had been transferred to the mitochondria. These genes were crucial for the photosynthetic process (Vrba and Curtis, 1990; Martín and Sabater, 2010; Berry et al., 2013), suggesting a possible correlation of unique environmental adaptation in *Opuntia* (Szarek et al., 1973; Mallona et al., 2011). Currently, there was no evidence of expression or functional regulation of these chloroplast genes in the mitochondria. However, following integration, these genes might become non-functional pseudogenes due to genetic recombination.

### 4.4 RNA editing events are prevalent in the PCGs of the *O. cochienllifora* mitogenome

RNA editing, a crucial post-transcriptional regulatory mechanism in higher plant organelles, produced transcripts that differ from the DNA template, predominantly through C-to-U base conversions (Edera et al., 2018; Hao et al., 2021). This process, mediated by various mechanisms and pathways (Hao et al., 2021), could modify organellar transcription products’ coding sequences, often creating translatable mRNAs by forming AUG start codons or removing premature termination codons (Edera et al., 2018; Small et al., 2020). In our study, all 33 protein-coding genes of the *O. cochienllifora* mitochondrial genome exhibited putative RNA editing sites, primarily single-base (C to U) edits leading to amino acid changes, potentially endowing these genes with novel structures and functions (Møller et al., 2021). Previous research had linked RNA editing to protein function initiation and maintenance in various crops (Kadowaki et al., 1995; Quiñones et al., 1995; Gray, 2003). Typically, the generation of new start and stop codons results in proteins that were more conserved and exhibit higher homology with counterparts from other species, enhancing mitochondrial gene expression (Edera et al., 2018). Our findings also indicated that RNA editing events in the *O. cochienllifora* mitochondrial genome generated start or stop codons in five genes: new start codons at *nad4L*-2, *nad7*-224, and *cox2*-443, and new stop codons at *atp9-copy*3-223 and *ccmFC*-1306. Notably, the *atp9* gene undergoes varying degrees of RNA editing across different crops, a process deemed essential for producing functional polypeptides (Wintz and Hanson, 1991). A specific editing site in the *ccmFC* gene was believed to be associated with regulation under salinity stress (Ramadan et al., 2023). However, the implications of these edits for mitochondrial function and overall plant physiology warrant further investigation.

## 5 Conclusion

This is the first published assembly of mitochondrial genome in the *Opuntia* genus, spanning 1,156,235 base pairs and encoding 54 unique genes. We identified the presence of dispersed repeats, fragments of plastid DNA, and RNA editing events with this genome, along with the potential for multiple structural conformations. Synteny and evolutionary analysis suggest frequent genomic recombination in the *O. cochenillifera* mitogenome. These findings offer crucial insights for comprehensive studies into the mitochondrial genetics of *Opuntia* and molecular breeding in these clonally reproductive species.

## Data availability statement

The datasets presented in this study can be found in online repositories. The names of the repository/repositories and accession number(s) can be found below: GenBank, OR885584 and OR885585.

## Author contributions

JL: Formal Analysis, Software, Visualization, Writing – original draft, Writing – review & editing, Data curation, Investigation, Methodology. YF: Methodology, Writing – review & editing. CC: Writing – review & editing, Methodology. JYa: Methodology, Writing – review & editing. XB: Methodology, Writing – review & editing. HL: Writing – review & editing, Methodology. CL: Methodology, Resources, Writing – review & editing. YX: Writing – original draft, Methodology. WT: Resources, Writing – original draft, Methodology. ZQ: Conceptualization, Data curation, Formal Analysis, Funding acquisition, Writing – original draft, Writing – review & editing, Methodology, Project administration, Supervision, Validation. JYu: Conceptualization, Methodology, Writing – review & editing, Project administration, Supervision, Validation. XY: Conceptualization, Data curation, Funding acquisition, Investigation, Project administration, Writing – original draft, Writing – review & editing, Supervision, Validation.

## Supplementary material

Supplementary Figure 1 The putative circular chloroplast genome maps of *O. cochenillifera*.

Supplementary Figure 2 The number of unique and common SSRs detected in mitochondrial genome of four Cactaceae species. Different colors represent different SSRs types.

Supplementary Figure 3 Frequency of identified SSRs types (Mono-, Di-. Tri-, Tetra, Penta- and Hexa- nucleotide repeats) detected in mitochondrial genome of four species of the Cactaceae family. Each column represents a different repeat type.

Supplementary Figure 4 Comparative analysis of mitochondrial genome repeat sequences in four species of Cactaceae.

Supplementary Table 8 Relative synonymous codon usage of each amino acid pair codon in the mitochondrial genome of *O. cochenillifera.*

## References

[B1] Almeida I. V. B. Rego M. M. Batista F. R. C. Araújo J. S. Souza J. T. A. Medeiros L. T. V. (2022). “Genetic improvement of *Opuntia* spp. for forage production in the Brazilian semi-arid region,” in Acta Horticulturae (ISHS, João Pessoa, Paraıíba, Brazil), 31–38. doi: 10.17660/actahortic.2022.1343.5

[B2] Alverson A. J. Rice D. W. Dickinson S. Barry K. Palmer J. D. (2011). Origins and recombination of the bacterial-sized multichromosomal mitochondrial genome of cucumber. Plant Cell 23, 2499–2513. doi: 10.1105/tpc.111.087189 21742987 PMC3226218

[B3] Anaya-Pérez M. A. (2001). “History of the use of *Opuntia* as forage in Mexico,” in Cactus *Opuntia* spp forage (FAO, Italy), 5–12. Available at: https://www.fao.org/3/Y2808E/y2808e05.htm#TopOfPage.

[B4] Andre C. Levy A. Walbot V. (1992). Small repeated sequences and the structure of plant mitochondrial genomes. Trends Genet. 8, 128–132. doi: 10.1016/0168-9525(92)90370-J 1631955

[B5] Arimura S. Ayabe H. Sugaya H. Okuno M. Tamura Y. Tsuruta Y.. (2020). Targeted gene disruption of ATP synthases 6-1 and 6-2 in the mitochondrial genome of *Arabidopsis thaliana* by mitoTALENs. Plant J. 104, 1459–1471. doi: 10.1111/tpj.15041 33098708

[B6] Arseneau J.-R. Steeves R. Laflamme M. (2017). Modified low-salt CTAB extraction of high-quality DNA from contaminant-rich tissues. Mol. Ecol. Resour. 17, 686–693. doi: 10.1111/1755-0998.12616 27768249

[B7] Asker S. Jerling L. (1992). Apomixis in plants (Boca Raton: CRC press).

[B8] Barba F. J. Putnik P. Kovačević D. B. Poojary M. M. Roohinejad S. Lorenzo J. M.. (2017). Impact of conventional and non-conventional processing on prickly pear (*Opuntia* spp.) and their derived products: From preservation of beverages to valorization of by-products. Trends Food Sci. Technol. 67, 260–270. doi: 10.1016/j.tifs.2017.07.012

[B9] Beier S. Thiel T. Münch T. Scholz U. Mascher M. (2017). MISA-web: a web server for microsatellite prediction. Bioinformatics 33, 2583–2585. doi: 10.1093/bioinformatics/btx198 28398459 PMC5870701

[B10] Benson G. (1999). Tandem repeats finder: a program to analyze DNA sequences. Nucleic Acids Res. 27, 573–580. doi: 10.1093/nar/27.2.573 9862982 PMC148217

[B11] Berry J. O. Yerramsetty P. Zielinski A. M. Mure C. M. (2013). Photosynthetic gene expression in higher plants. Photosynth. Res. 117, 91–120. doi: 10.1007/s11120-013-9880-8 23839301

[B12] Carra A. Catalano C. Pathirana R. Sajeva M. Inglese P. Motisi A.. (2023). Increased Zygote-Derived Plantlet Formation through *In Vitro* Rescue of Immature Embryos of Highly Apomictic *Opuntia ficus-indica* (Cactaceae). Plants 12, 2758. doi: 10.3390/plants12152758 37570913 PMC10421068

[B13] Chen Y. Ye W. Zhang Y. Xu Y. (2015). High speed BLASTN: an accelerated MegaBLAST search tool. Nucleic Acids Res. 43, 7762–7768. doi: 10.1093/nar/gkv784 26250111 PMC4652774

[B14] Christensen A. C. (2013). Plant mitochondrial genome evolution can be explained by DNA repair mechanisms. Genome Biol. Evol. 5, 1079–1086. doi: 10.1093/gbe/evt069 23645599 PMC3698917

[B15] Cole L. W. Guo W. Mower J. P. Palmer J. D. (2018). High and variable rates of repeat-mediated mitochondrial genome rearrangement in a genus of plants. Mol. Biol. Evol. 35, 2773–2785. doi: 10.1093/molbev/msy176 30202905

[B16] Copetti D. Búrquez A. Bustamante E. Charboneau J. L. Childs K. L. Eguiarte L. E.. (2017). Extensive gene tree discordance and hemiplasy shaped the genomes of North American columnar cacti. Proc. Natl. Acad. Sci. 114, 12003–12008. doi: 10.1073/pnas.1706367114 29078296 PMC5692538

[B17] Duminil J. Besnard G. (2021). “Utility of the mitochondrial genome in plant taxonomic studies,” in Molecular plant taxonomy: Methods and protocols (Springer US, New York, NY), 107–118. doi: 10.1007/978-1-0716-0997-2_6 33301090

[B18] Edera A. A. Gandini C. L. Sanchez-Puerta M. V. (2018). Towards a comprehensive picture of C-to-U RNA editing sites in angiosperm mitochondria. Plant Mol. Biol. 97, 215–231. doi: 10.1007/s11103-018-0734-9 29761268

[B19] Edera A. A. Small I. Milone D. H. Sanchez-Puerta M. V. (2021). Deepred-Mt: Deep representation learning for predicting C-to-U RNA editing in plant mitochondria. Comput. Biol. Med. 136, 104682. doi: 10.1016/j.compbiomed.2021.104682 34343887

[B20] Forner J. Kleinschmidt D. Meyer E. H. Fischer A. Morbitzer R. Lahaye T.. (2022). Targeted introduction of heritable point mutations into the plant mitochondrial genome. Nat. Plants 8, 245–256. doi: 10.1038/s41477-022-01108-y 35301443 PMC8940627

[B21] Galtier N. Nabholz B. Glémin S. Hurst G. D. D. (2009). Mitochondrial DNA as a marker of molecular diversity: a reappraisal. Mol. Ecol. 18, 4541–4550. doi: 10.1111/j.1365-294X.2009.04380.x 19821901

[B22] Gao C. W. Wu C. H. Zhang Q. Zhao X. Wu M. X. Chen R. R. (2020). Characterization of chloroplast genomes from two salvia medicinal plants and gene transfer among their mitochondrial and chloroplast genomes. Front. Genet. 11, 574962. doi: 10.3389/fgene.2020.574962 33193683 PMC7642825

[B23] Gentile A. La Malfa S. (2022). “Needs and strategies for breeding and sustainable use of genetic resources in *Opuntia* ,” in Acta Horticulturae (ISHS, João Pessoa, Paraıíba, Brazil), 31–38. doi: 10.17660/ActaHortic.2022.1343.5

[B24] Ghulam M. M. Kousar S. Vardhan H. (2015). “Plant mitochondrial omics: State-of-the-art knowledge,” in PlantOmics: The omics of plant science. eds. Barh D. Khan M. S. Davies E. (New Delhi: Springer India), 573–613. doi: 10.1007/978-81-322-2172-2_20

[B25] Gray M. (2003). Diversity and evolution of mitochondrial RNA editing systems. IUBMB Life 55, 227–233. doi: 10.1080/1521654031000119425 12880203

[B26] Gualberto J. M. Newton K. J. (2017). Plant mitochondrial genomes: dynamics and mechanisms of mutation. Annu. Rev. Plant Biol. 68, 225–252. doi: 10.1146/annurev-arplant-043015-112232 28226235

[B27] Handa H. (2003). The complete nucleotide sequence and RNA editing content of the mitochondrial genome of rapeseed (*Brassica napus* L.): comparative analysis of the mitochondrial genomes of rapeseed and *Arabidopsis thaliana* . Nucleic Acids Res. 31, 5907–5916. doi: 10.1093/nar/gkg795 14530439 PMC219474

[B28] Hao W. Liu G. Wang W. Shen W. Zhao Y. Sun J.. (2021). RNA editing and its roles in plant organelles. Front. Genet. 12. doi: 10.3389/fgene.2021.757109 PMC8511385 34659369

[B29] Hernández-Hernández T. Brown J. W. Schlumpberger B. O. Eguiarte L. E. Magallón S. (2014). Beyond aridification: multiple explanations for the elevated diversification of cacti in the New World Succulent Biome. New Phytol. 202, 1382–1397. doi: 10.1111/nph.12752 24611540

[B30] Hojsgaard D. Hörandl E. (2019). The rise of apomixis in natural plant populations. Front. Plant Sci. 10. doi: 10.3389/fpls.2019.00358 PMC6454013 31001296

[B31] Jorge A. O. Costa A. S. Oliveira M. B. P. (2023). Adapting to climate change with *Opuntia* . Plants 12, 2907. doi: 10.3390/plants12162907 37631119 PMC10457962

[B32] Kadowaki K. Ozawa K. Kazama S. Kubo N. Akihama T. (1995). Creation of an initiation codon by RNA editing in the coxl transcript from tomato mitochondria. Curr. Genet. 28, 415–422. doi: 10.1007/BF00310809 8575013

[B33] Kang B.-C. Bae S.-J. Lee S. Lee J. S. Kim A. Lee H.. (2021). Chloroplast and mitochondrial DNA editing in plants. Nat. Plants 7, 899–905. doi: 10.1038/s41477-021-00943-9 34211132 PMC8289734

[B34] Katoh K. Standley D. M. (2013). MAFFT multiple sequence alignment software version 7: improvements in performance and usability. Mol. Biol. Evol. 30, 772–780. doi: 10.1093/molbev/mst010 23329690 PMC3603318

[B35] Kazama T. Okuno M. Watari Y. Yanase S. Koizuka C. Tsuruta Y.. (2019). Curing cytoplasmic male sterility *via* TALEN-mediated mitochondrial genome editing. Nat. Plants 5, 722–730. doi: 10.1038/s41477-019-0459-z 31285556

[B36] Khachaturyan M. Reusch T. B. Dagan T. (2023). Worldwide population genomics reveal long-term stability of the mitochondrial genome architecture in a keystone marine plant. Genome Biol. Evol. 15, evad167. doi: 10.1093/gbe/evad167 37708410 PMC10538256

[B37] Knoop V. (2012). Seed plant mitochondrial genomes: complexity evolving. Genomics Chloroplasts Mitochondria 35, 175–200. doi: 10.1007/978-94-007-2920-9_8

[B38] Kondo A. Ito M. Takeda Y. Kurahashi Y. Toh S. Funaguma T. (2023). Morphological and antioxidant responses of *Nopalea cochenillifera* cv. Maya (edible *Opuntia* sp.”Kasugai Saboten”) to chilling acclimatization. J. Plant Res. 136, 211–225. doi: 10.1007/s10265-023-01437-9 36690846 PMC9988806

[B39] Kubo T. Newton K. J. (2008). Angiosperm mitochondrial genomes and mutations. Mitochondrion 8, 5–14. doi: 10.1016/j.mito.2007.10.006 18065297

[B40] Kubo T. Nishizawa S. Sugawara A. Itchoda N. Estiati A. Mikami T. (2000). The complete nucleotide sequence of the mitochondrial genome of sugar beet (*Beta vulgaris* L.) reveals a novel gene for tRNACys (GCA). Nucleic Acids Res. 28, 2571–2576. doi: 10.1093/nar/28.13.2571 10871408 PMC102699

[B41] Kumar S. Stecher G. Tamura K. (2016). MEGA7: molecular evolutionary genetics analysis version 7.0 for bigger datasets. Mol. Biol. Evol. 33, 1870–1874. doi: 10.1093/molbev/msw054 27004904 PMC8210823

[B42] Kurtz S. Choudhuri J. V. Ohlebusch E. Schleiermacher C. Stoye J. Giegerich R. (2001). REPuter: the manifold applications of repeat analysis on a genomic scale. Nucleic Acids Res. 29, 4633–4642. doi: 10.1093/nar/29.22.4633 11713313 PMC92531

[B43] Landrum J. V. (2002). Four succulent families and 40 million years of evolution and adaptation to xeric environments: What can stem and leaf anatomical characters tell us about their phylogeny? Taxon 51, 463–473. doi: 10.2307/1555064

[B44] Lans C. A. (2006). Ethnomedicines used in Trinidad and Tobago for urinary problems and diabetes mellitus. J. Ethnobiol. Ethnomed. 2, 45. doi: 10.1186/1746-4269-2-45 17040567 PMC1624823

[B45] Lassalle F. Périan S. Bataillon T. Nesme X. Duret L. Daubin V. (2015). GC-content evolution in bacterial genomes: the biased gene conversion hypothesis expands. PloS Genet. 11, e100494. doi: 10.1371/journal.pgen.1004941 PMC4450053 25659072

[B46] Letunic I. Bork P. (2019). Interactive Tree Of Life (iTOL) v4: recent updates and new developments. Nucleic Acids Res. 47, W256–W259. doi: 10.1093/nar/gkz239 30931475 PMC6602468

[B47] Lewis S. E. Searle S. M. J. Harris N. Gibson M. Iyer V. Richter J.. (2002). Apollo: a sequence annotation editor. Genome Biol. 3, 1–14. doi: 10.1186/gb-2002-3-12-research0082 PMC151184 12537571

[B48] Li H. (2013). Aligning sequence reads, clone sequences and assembly contigs with BWA-MEM. Preprint at https://arxiv.org/abs/1303.399.

[B49] Li H. Durbin R. (2009). Fast and accurate short read alignment with Burrows–Wheeler transform. Bioinformatics 25, 1754–1760. doi: 10.1093/bioinformatics/btp324 19451168 PMC2705234

[B50] Liu S. Ni Y. Li J. Zhang X. Yang H. Chen H.. (2023). CPGView: a package for visualizing detailed chloroplast genome structures. Mol. Ecol. Resour. 23, 694–704. doi: 10.1111/1755-0998.13729 36587992

[B51] Lowe T. M. Eddy S. R. (1997). tRNAscan-SE: a program for improved detection of transfer RNA genes in genomic sequence. Nucleic Acids Res. 25, 955–964. doi: 10.1093/nar/25.5.955 9023104 PMC146525

[B52] Lu G. Wang W. Mao J. Li Q. Que Y. (2023). Complete mitogenome assembly of *Selenicereus monacanthus* revealed its molecular features, genome evolution, and phylogenetic implications. BMC Plant Biol. 23, 541. doi: 10.1186/s12870-023-04529-9 37924024 PMC10625231

[B53] Majure L. C. Puente-Martinez R. (2014). Phylogenetic relationships and morphological evolution in *Opuntia* s.str and closely related members of tribe Opuntieae. Succul. Plant Res. 8, 9–30.

[B54] Maliga P. (2022). Engineering the plastid and mitochondrial genomes of flowering plants. Nat. Plants 8, 996–1006. doi: 10.1038/s41477-022-01227-6 36038655

[B55] Mallona I. Egea-Cortines M. Weiss J. (2011). Conserved and divergent rhythms of crassulacean acid metabolism-related and core clock gene expression in the cactus *Opuntia ficus-indica* . Plant Physiol. 156, 1978–1989. doi: 10.1104/pp.111.179275 21677095 PMC3149932

[B56] Mandujano M. C. Golubov J. Huenneke L. F. (2007). Effect of reproductive modes and environmental heterogeneity in the population dynamics of a geographically widespread clonal desert cactus. Popul. Ecol. 49, 141–153. doi: 10.1007/s10144-006-0032-2

[B57] Martín M. Sabater B. (2010). Plastid *ndh* genes in plant evolution. Plant Physiol. Biochem. 48, 636–645. doi: 10.1016/j.plaphy.2010.04.009 20493721

[B58] May R. A. Stevenson K. J. (2009). Software review of origin 8. J. Am. Chem. Soc 131, 872–872. doi: 10.1021/ja809638x

[B59] Mehrotra S. Goyal V. (2014). Repetitive sequences in plant nuclear DNA: types, distribution, evolution and function. Genomics Proteomics Bioinf. 12, 164–171. doi: 10.1016/j.gpb.2014.07.003 PMC4411372 25132181

[B60] Minh B. Q. Schmidt H. A. Chernomor O. Schrempf D. Woodhams M. D. Von Haeseler A.. (2020). IQ-TREE 2: new models and efficient methods for phylogenetic inference in the genomic era. Mol. Biol. Evol. 37, 1530–1534. doi: 10.1093/molbev/msaa015 32011700 PMC7182206

[B61] Møller I. M. Rasmusson A. G. Van Aken O. (2021). Plant mitochondria – past, present and future. Plant J. 108, 912–959. doi: 10.1111/tpj.15495 34528296

[B62] Mower J. P. Sloan D. B. Alverson A. J. (2012). “Plant mitochondrial genome diversity: the genomics revolution,” in Plant genome diversity volume 1: Plant genomes, their residents, and their evolutionary dynamics. Eds. Wendel J. F. Greilhuber J. Dolezel J. Leitch I. J. (Vienna: Springer), 123–144. doi: 10.1007/978-3-7091-1130-7_9

[B63] Notsu Y. Masood S. Nishikawa T. Kubo N. Akiduki G. Nakazono M.. (2002). The complete sequence of the rice (*Oryza sativa* L.) mitochondrial genome: frequent DNA sequence acquisition and loss during the evolution of flowering plants. Mol. Genet. Genomics 268, 434–445. doi: 10.1007/s00438-002-0767-1 12471441

[B64] Ogihara Y. Yamazaki Y. Murai K. Kanno A. Terachi T. Shiina T.. (2005). Structural dynamics of cereal mitochondrial genomes as revealed by complete nucleotide sequencing of the wheat mitochondrial genome. Nucleic Acids Res. 33, 6235–6250. doi: 10.1093/nar/gki925 16260473 PMC1275586

[B65] Plancarte D. C. Solórzano S. (2023). Structural and gene composition variation of the complete mitochondrial genome of *Mammillaria huitzilopochtli* (Cactaceae, Caryophyllales), revealed by *de novo* assembly. BMC Genomics 24, 509. doi: 10.1186/s12864-023-09607-8 37653379 PMC10468871

[B66] Prisa D. (2023). *Opuntia* plants nutritive and medicinal value: a review. J. Curr. Sci. Technol. 13, 486–499. doi: 10.59796/jcst.V13N2.2023.1762

[B67] Quiñones V. Zanlungo S. Holuigue L. Litvak S. Jordana X. (1995). The *cox1* initiation codon is created by RNA editing in potato mitochondria. Plant Physiol. 108, 1327. doi: 10.1104/pp.108.3.1327 7630963 PMC157503

[B68] Ramadan A. Alnufaei A. A. Fiaz S. Khan T. K. Hassan S. M. (2023). Effect of salinity on ccmfn gene RNA editing of mitochondria in wild barley and uncommon types of RNA editing. Funct. Integr. Genomics 23, 50. doi: 10.1007/s10142-023-00978-5 36707470

[B69] Ramadan M. F. Ayoub T. E. M. Rohn S. (Eds.) (2021). *Opuntia* spp: *Chemistry, Bioactivity and Industrial Applications* (Cham: Springer International Publishing). doi: 10.1007/978-3-030-78444-7

[B70] Russell C. E. Felker P. (1987). The prickly-pears (*Opuntia* spp., Cactaceae): A source of human and animal food in semiarid regions. Econ. Bot. 41, 433–445. doi: 10.1007/BF02859062

[B71] Shi L. Chen H. Jiang M. Wang L. Wu X. Huang L.. (2019). CPGAVAS2, an integrated plastome sequence annotator and analyzer. Nucleic Acids Res. 47, 1978–1989. doi: 10.1093/nar/gkz345 PMC6602467 31066451

[B72] Small I. D. Schallenberg-Rüdinger M. Takenaka M. Mireau H. Ostersetzer-Biran O. (2020). Plant organellar RNA editing: what 30 years of research has revealed. Plant J. 101, 1040–1056. doi: 10.1111/tpj.14578 31630458

[B73] Sperisen C. Büchler U. Gugerli F. Mátyás G. Geburek T. Vendramin G. G. (2001). Tandem repeats in plant mitochondrial genomes: application to the analysis of population differentiation in the conifer Norway spruce. Mol. Ecol. 10, 257–263. doi: 10.1046/j.1365-294X.2001.01180.x 11251804

[B74] Szarek S. R. Johnson H. B. Ting I. P. (1973). Drought adaptation in *Opuntia basilaris*: significance of recycling carbon through Crassulacean acid metabolism. Plant Physiol. 52, 539–541. doi: 10.1104/pp.52.6.539 16658600 PMC366540

[B75] Takatsuka A. Kazama T. Arimura S. Toriyama K. (2022). TALEN-mediated depletion of the mitochondrial gene *orf312* proves that it is a Tadukan-type cytoplasmic male sterility-causative gene in rice. Plant J. 110, 994–1004. doi: 10.1111/tpj.15715 35218074

[B76] Tang H. Zheng X. Li C. Xie X. Chen Y. Chen L.. (2017). Multi-step formation, evolution, and functionalization of new cytoplasmic male sterility genes in the plant mitochondrial genomes. Cell Res. 27, 130–146. doi: 10.1038/cr.2016.115 27725674 PMC5223224

[B77] Tillich M. Lehwark P. Pellizzer T. Ulbricht-Jones E. S. Fischer A. Bock R.. (2017). GeSeq–versatile and accurate annotation of organelle genomes. Nucleic Acids Res. 45, W6–W11. doi: 10.1093/nar/gkx391 28486635 PMC5570176

[B78] Trávníček P. Čertner M. Ponert J. Chumová Z. Jersáková J. Suda J. (2019). Diversity in genome size and GC content shows adaptive potential in orchids and is closely linked to partial endoreplication, plant life-history traits and climatic conditions. New Phytol. 224, 1642–1656. doi: 10.1111/nph.15996 31215648

[B79] Vrba J. M. Curtis S. E. (1990). Characterization of a four-member *psb* A gene family from the cyanobacterium *Anabaena* PCC 7120. Plant Mol. Biol. 14, 81–92. doi: 10.1007/BF00015657 2129283

[B80] Wang Y. Tang H. DeBarry J. D. Tan X. Li J. Wang X.. (2012). MCScanX: a toolkit for detection and evolutionary analysis of gene synteny and collinearity. Nucleic Acids Res. 40, e49–e49. doi: 10.1093/nar/gkr1293 22217600 PMC3326336

[B81] Wang D. Wu Y.-W. Shih A. C.-C. Wu C.-S. Wang Y.-N. Chaw S.-M. (2007). Transfer of chloroplast genomic DNA to mitochondrial genome occurred at least 300 MYA. Mol. Biol. Evol. 24, 2040–2048. doi: 10.1093/molbev/msm133 17609537

[B82] Wang Z. Xie L. Prather C. M. Guo H. Han G. Ma C. (2018). What drives the shift between sexual and clonal reproduction of *Caragana stenophylla* along a climatic aridity gradient? BMC Plant Biol. 18, 1–10. doi: 10.1186/s12870-018-1313-6 29788911 PMC5964679

[B83] Whelan J. Murcha M. W. (2015). Plant mitochondria: methods and protocols (Humana New York, NY: Springer Science). doi: 10.1007/978-1-4939-2639-8

[B84] Wick R. R. Judd L. M. Gorrie C. L. Holt K. E. (2017). Unicycler: resolving bacterial genome assemblies from short and long sequencing reads. PloS Comput. Biol. 13, e1005595. doi: 10.1371/journal.pcbi.1005595 28594827 PMC5481147

[B85] Wick R. R. Schultz M. B. Zobel J. Holt K. E. (2015). Bandage: interactive visualization of *de novo* genome assemblies. Bioinformatics 31, 3350–3352. doi: 10.1093/bioinformatics/btv383 26099265 PMC4595904

[B86] Wintz H. Hanson M. R. (1991). A termination codon is created by RNA editing in the petunia mitochondrial *atp* 9 gene transcript. Curr. Genet. 19, 61–64. doi: 10.1007/BF00362089 1709830

[B87] Wynn E. L. Christensen A. C. (2019). Repeats of unusual size in plant mitochondrial genomes: identification, incidence and evolution. G3 Genes, Genomes, Genetics 9, 549–559. doi: 10.1534/g3.118.200948 30563833 PMC6385970

[B88] Xiong Y. Yu Q. Xiong Y. Zhao J. Lei X. Liu L.. (2022). The complete mitogenome of *Elymus sibiricus* and insights into its evolutionary pattern based on simple repeat sequences of seed plant mitogenomes. Front. Plant Sci. 12. doi: 10.3389/fpls.2021.802321 PMC8826237 35154192

[B89] Zhang D. Gao F. Jakovlić I. Zou H. Zhang J. Li W. X.. (2020). PhyloSuite: An integrated and scalable desktop platform for streamlined molecular sequence data management and evolutionary phylogenetics studies. Mol. Ecol. Resour. 20, 348–355. doi: 10.1111/1755-0998.13096 31599058

[B90] Zhang H. Meltzer P. Davis S. (2013). RCircos: an R package for Circos 2D track plots. BMC Bioinf. 14, 1–5. doi: 10.1186/1471-2105-14-244 PMC3765848 23937229

[B91] Zhang X. Shan Y. Li J. Qin Q. Yu J. Deng H. (2023). Assembly of the complete mitochondrial genome of *Pereskia aculeata* revealed that two pairs of repetitive elements mediated the recombination of the genome. Int. J. Mol. Sci. 24, 8366. doi: 10.3390/ijms24098366 37176072 PMC10179450

