# Supplementary figures and images for: Genomic insights into the clonal reproductive Opuntia cochenillifera: mitochondrial and chloroplast genomes of the cochineal cactus for enhanced understanding of structural dynamics and evolutionary implications
Source: Front Plant Sci. 2024 Mar 7;15:1347945. doi: 10.3389/fpls.2024.1347945 (PMC10954886; doi:10.3389/fpls.2024.1347945)

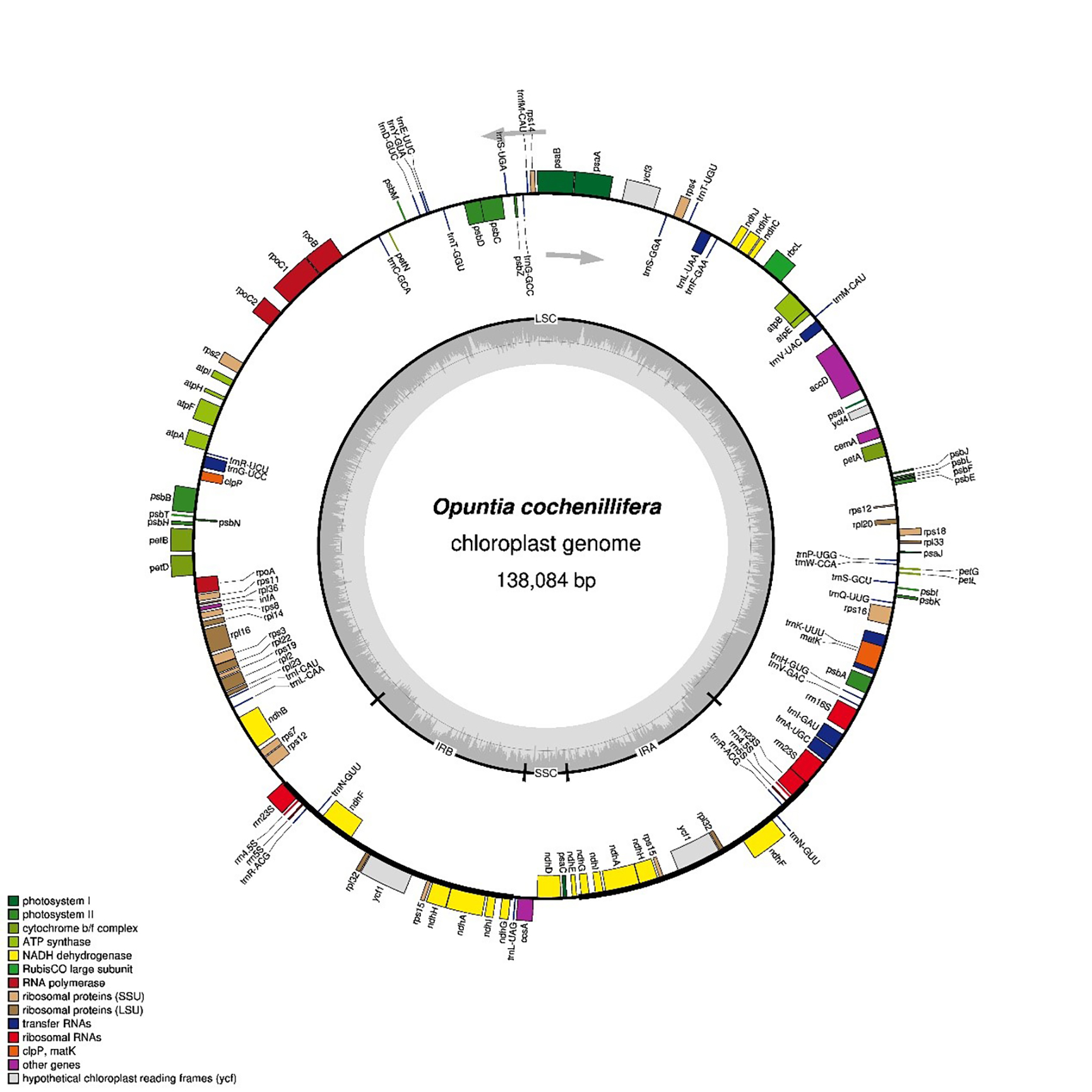

Supplement: Supplementary Figure 1 — The putative circular chloroplast genome maps of O. cochenillifera. [file Image_1.jpeg]

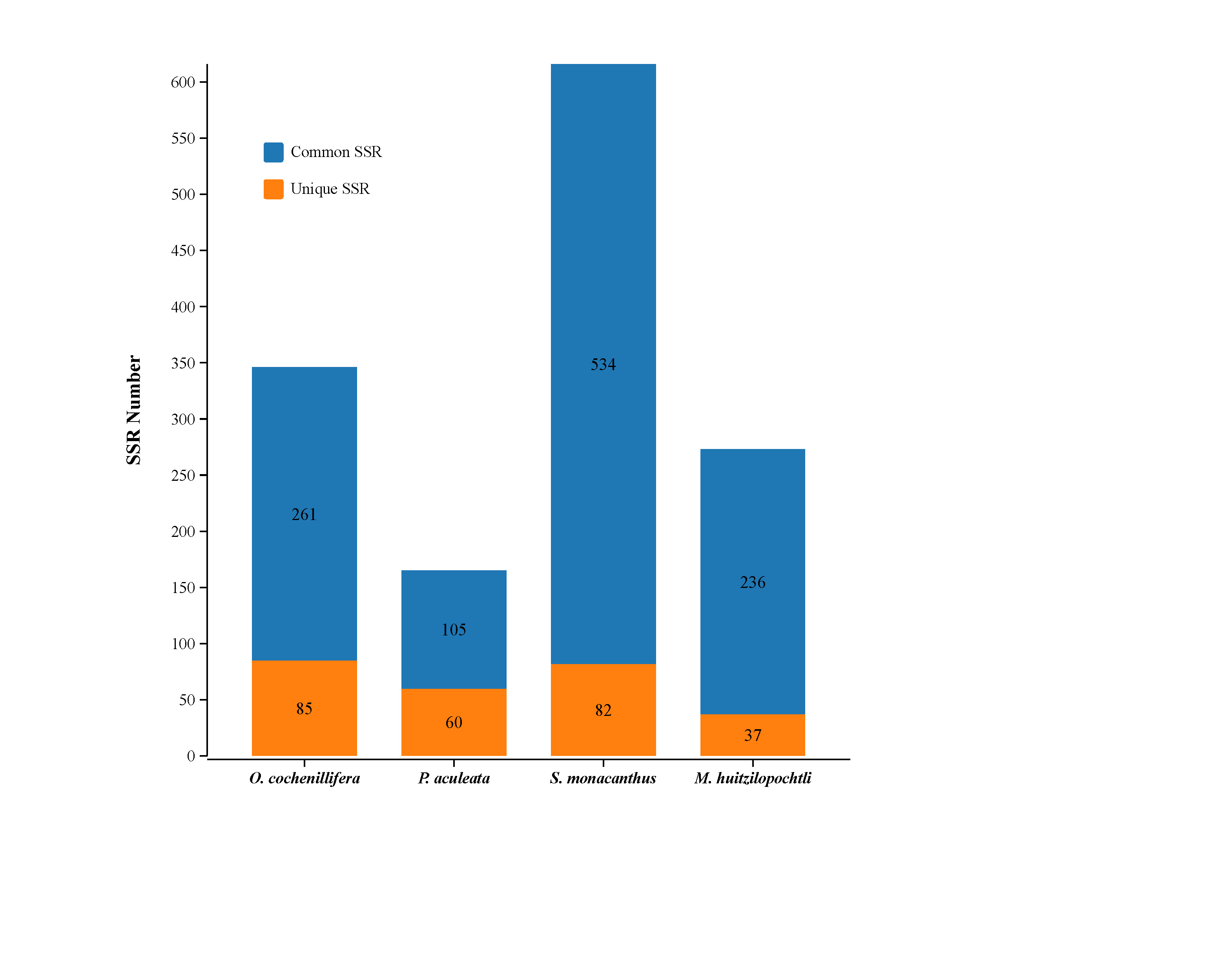

Supplement: Supplementary Figure 2 — The number of unique and common SSRs detected in mitochondrial genome of four Cactaceae species. Different colors represent different SSRs types. [file Image_2.jpeg]

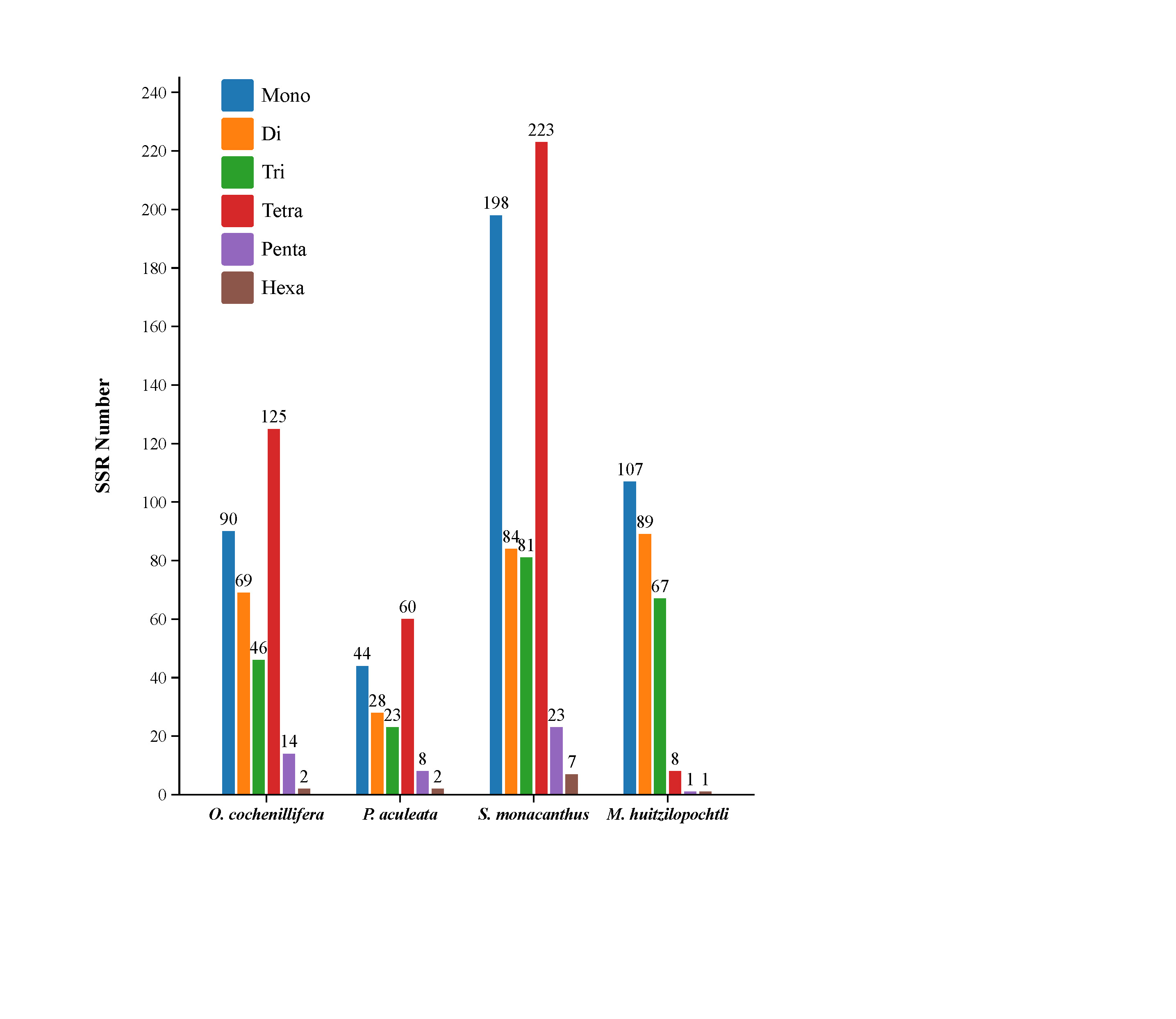

Supplement: Supplementary Figure 3 — Frequency of identified SSRs types (Mono-, Di-. Tri-, Tetra, Penta- and Hexa- nucleotide repeats) detected in mitochondrial genome of four species of the Cactaceae family. Each column represents a different repeat type. [file Image_3.jpeg]

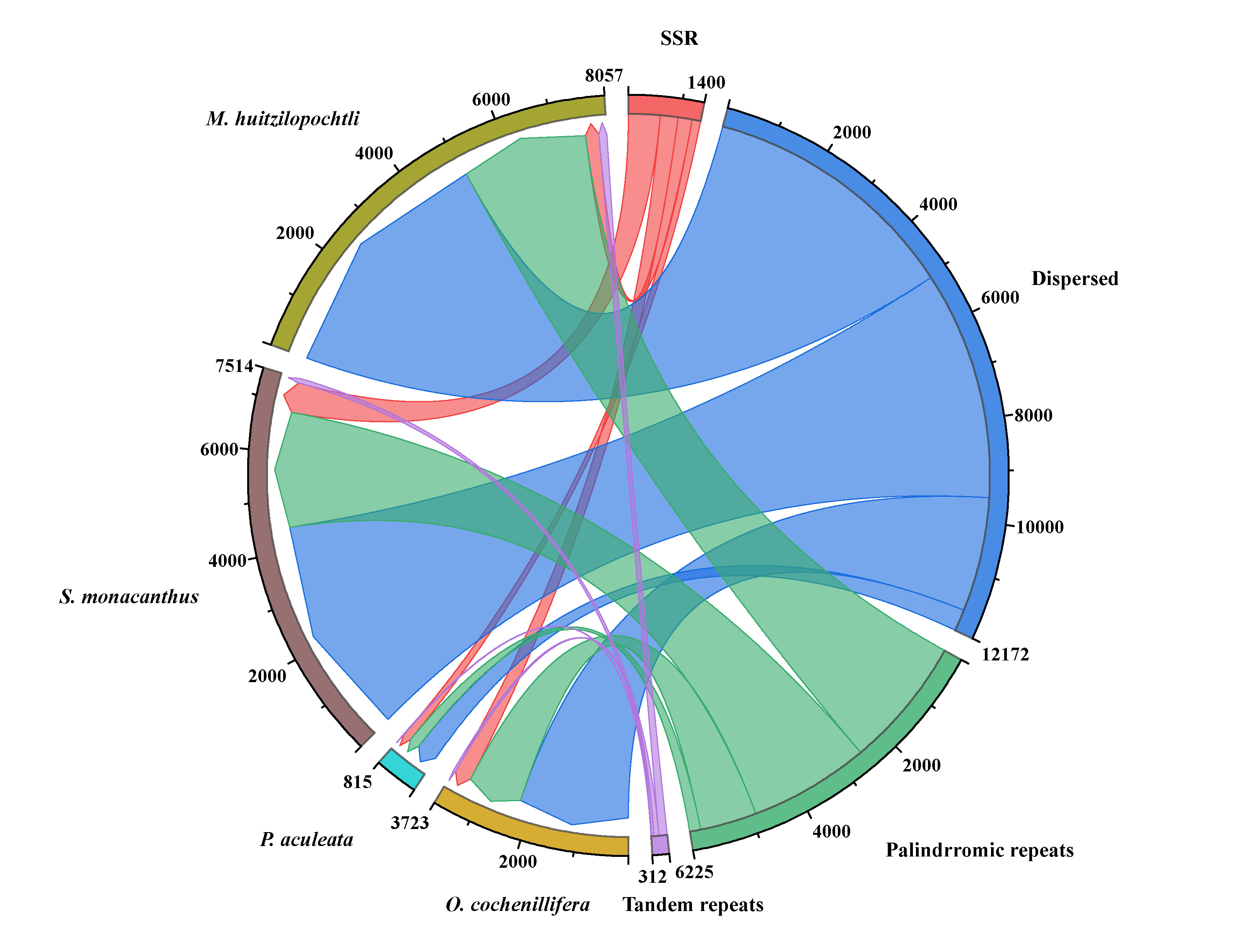

Supplement: Supplementary Figure 4 — Comparative analysis of mitochondrial genome repeat sequences in four species of Cactaceae. [file Image_4.jpeg]
